# NOTCH1 signaling contributes to cell growth, anti-apoptosis and metastasis in salivary adenoid cystic carcinoma

**DOI:** 10.18632/oncotarget.2321

**Published:** 2014-08-06

**Authors:** Bo-Hua Su, Jing Qu, Min Song, Xiao-Yu Huang, Xiao-Meng Hu, Jian Xie, Yong Zhao, Lin-Can Ding, Lin She, Jiang Chen, Li-Song Lin, Xu Lin, Da-Li Zheng, You-Guang Lu

**Affiliations:** ^1^ Department of Preventive Dentistry, Affiliated Stomatological Hospital, Fujian Medical University, Fuzhou, China; ^2^ Department of Pathology, Affiliated Stomatological Hospital, Fujian Medical University, Fuzhou, China; ^3^ Center of Dental Implant, Affiliated Stomatological Hospital, Fujian Medical University, Fuzhou, China; ^4^ Department of Oral and Maxillofacial Surgery, Affiliated First Hospital of Fujian Medical University, Fuzhou, China; ^5^ Key Laboratory of Ministry of Education for Gastrointestinal Cancer, School of Basic Medical Sciences, Fujian Medical University, Fuzhou, China; ^6^ Molecular Oncology, Moffitt Cancer Center, Tampa, FL, United States

**Keywords:** NOTCH1, salivary adenoid cystic carcinoma, proliferation, apoptosis, metastasis

## Abstract

Background: Numerous studies have reported both the tumor-suppressive and oncogenic roles of the Notch pathway, indicating that Notch activity regulates tumor biology in a complex, context-dependent manner. The aim of the present study was to identify the role of NOTCH1 in the cell growth and metastasis of SACC.

Methods: We analyzed the expression of NOTCH1 in clinical SACC samples using immunohistochemical staining. We silenced the expression of NOTCH1 and overexpressed activated NOTCH1 to elucidate the effects of NOTCH1 on proliferation, migration and invasion. NOTCH1 target genes were validated by real-time PCR.

Results: Our results showed that NOTCH1 was upregulated in SACC tissues when compared with normal tissues, and this upregulation was further enhanced in SACC tissues with metastasis and recurrence when compared with SACC tissues without metastasis. Overexpression of NOTCH1 in SACC cells promoted cell growth, migration and invasion, and knockdown of NOTCH1 inhibited cell proliferation *in vitro* and tumorigenicity *in vivo* by inducing cell apoptosis.

Conclusions:The results of this study suggest that NOTCH1 plays a key role in the cell growth, anti-apoptosis, and metastasis of SACC. NOTCH1 inhibitors might therefore have potential therapeutic applications in treating SACC patients by inhibiting cancer cell growth and metastasis.

## INTRODUCTION

Adenoid cystic carcinoma (ACC) is an uncommon malignant neoplasm that arises within secretory glands, most commonly the major and minor salivary glands of the head and neck. The biological properties of this carcinoma include aggressive growth, nerve and blood vessel invasion, distant metastases and high rates of recurrence, which result in poor patient survival (http://www.oralcancerfoundation.org/facts/rare/ac/). In a study of a cohort of 160 ACC patients, disease-specific survival was 89% at 5 years but only 40% at 15 years [1]. Distant metastasis is the most common presentation of treatment failure. The lung is by far the most common site of metastasis, with the liver being the second most common. Therefore, many studies have focused on novel targeted therapies for adenoid cystic carcinoma of the salivary gland.

The Notch signaling pathway is highly conserved and regulates cell-fate decisions throughout embryonic development and adult life. In mammals, there are four Notch proteins (Notch1-4), all of which are trans-membrane receptors. Activation of the canonical Notch signaling pathway requires physical contact of the Notch receptor with a ligand from one of two families, Jagged (Jagged1, 2) and Delta-like ligand (DLL-1, 3, 4), whereas both the receptor and ligand are attached to their respective cell membranes [2,3]. Therefore, the interaction of the Notch receptor and ligand occurs in two adjacent cells. After binding with their ligand, Notch receptors undergo a series of proteolytic cleavages, resulting in the release of the Notch intracellular domain (NICD), which translocates to the nucleus, driving the expression of HEY1 [4], HES1 [5], MYC [6], CCND1 [7], BCL2 [8] and other genes that regulate multiple cellular processes ranging from proliferation, differentiation, and stem cell maintenance, apoptosis.

In the process of malignant tumor formation, a variety of microenvironments interact: these microenvironments include immune cells and vascular endothelial cells, among others. The Notch signaling pathway can link these microenvironmental factors and thereby play an important role in tumor occurrence and development. The Notch pathway is genetically altered in a large number of hematopoietic and solid tumors, which can lead to either activation or repression of the pathway, depending on the context and the activation status of other oncogenic pathways. Both tumor-suppressive and oncogenic roles of the Notch pathway have been reported in multiple studies [2, 3]. As listed in a recent review [3], the Notch pathway demonstrated oncogenic roles in T cell acute lymphoblastic leukemia, chronic lymphocytic leukemia, colorectal cancer, lung adenocarcinoma, breast cancer, and prostate cancer; whereas tumor-suppressive roles were observed in B cell acute lymphoblastic leukemia, acute myeloid leukemia, small cell lung cancer, and squamous cell lung carcinoma. Even within the same tumor type, Notch may act as an oncogene or a tumor-suppressor gene. For example, Qi et al. reported that activated NOTCH1 signaling inhibits the growth of human liver cancer cells through the induction of cell cycle arrest and apoptosis, and the deletion of Notch1 in the liver of mice results in hyperproliferative hepatocytes [9], suggesting a tumor-suppressive role of Notch in liver cancer. However, studies from two independent groups have shown that liver-specific expression of the intracellular domain of Notch1 [10] or Notch2 [11] is sufficient to induce the formation of hepatocellular carcinoma in mice, and Notch may be important for the development of tumors following hepatitis B virus infection [12]. These studies demonstrated that Notch activity regulates tumor biology in a complex, context-dependent manner. The roles of the Notch pathway in human SACC remain unclear. Our previous study showed that NOTCH1, NOTCH3, and NOTCH4 were upregulated in ACC-M cells, an adenoid cystic carcinoma cell line with high metastatic potential, compared with ACC-2 cells, which have low metastatic potential, and knockdown of NOTCH4 in ACC-M cells inhibited the migratory and invasive abilities of the cells, indicating a pro-metastasis role of Notch4 in SACC [13].

In the present study, we analyzed the expression of NOTCH1 in clinical salivary adenoid cystic carcinoma samples using immunohistochemical staining. In an SACC cell line, we silenced the expression of NOTCH1 using small interfering RNA (siRNA) and overexpressed activated NOTCH1 using adenovirus carrying the NOTCH1 intracellular domain to elucidate the effects of NOTCH1 on the proliferation, migration and invasion of SACC.

## RESULTS

### NOTCH1 is upregulated in adenoid cystic carcinomas with metastasis and recurrence

**Figure 1:**
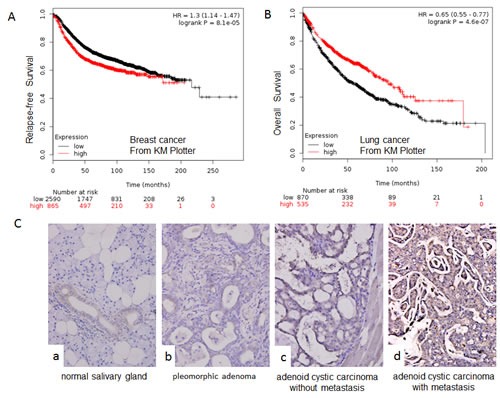
NOTCH1 is upregulated in salivary adenoid cystic carcinoma A-B, Survival of patients with breast cancer (A) or lung cancer (B) relative to different expression levels of NOTCH1 using KM Plotter from publicly available microarray data; C, Representative images for the expression of NOTCH1 by immunohistochemistry in normal salivary gland tissues (a), pleomorphic adenoma samples (b), and adenoid cystic carcinoma without (c) or with metastasis and recurrence (d) (Original magnification 400X).

Both the overexpression and downregulation of NOTCH1 have been observed in human cancers when compared with normal samples. In this study, we first explored the expression of NOTCH1 in different human cancers in the Oncomine database. According to this public available database, NOTCH1 is mainly overexpressed in brain cancer (Fig S1A-B) [15], gastric cancer (Fig S1A and C) [16], colorectal cancer, leukemia, and ovarian cancer. In contrast, NOTCH1 has also been shown to be downregulated in breast cancer (Fig S1A and D) [17], lung cancer Fig S1A and E) [18], prostate cancer, kidney cancer, and myeloma. Gene expression data for these studies are available through the Oncomine database, as are the studies describing these results (http://www.oncomine.org). We also conducted an unbiased bioinformatic analysis of gene-expression profiles of 3355 patients with breast cancer [19], 1405 with lung cancer [20] and 1171 with ovarian cancer [21] using Kaplan-Meier (KM) Plotter, a meta-analysis-based biomarker assessment tool. This analysis tool utilizes Affymetrix gene-expression profiling data, which involve multiple probe sets for most genes. The results showed that higher expression of NOTCH1 (auto select best cut-off) was associated with poor prognosis and shorter relapse-free survival (RFS; P = 8*10^−5^, Fig. 1A) in breast cancer but better prognosis and longer overall survival (OS; P=4.4*10^−7^, Fig. 1B) in lung cancer, with no significant difference in ovarian cancer (data not shown). In our previous study, we found that NOTCH1 was upregulated in the highly metastatic adenoid cystic carcinoma cell line, ACC-M, compared with the low metastatic cell line, ACC-2, suggesting that NOTCH1 contributes to the metastasis of adenoid cystic carcinoma. Next, we investigated the expression of NOTCH1 in salivary adenoid cystic carcinoma using immunohistochemistry. As shown in Fig. 1C and Table 1, NOTCH1 expression was absent or very low in normal salivary gland tissues (except in the ductal cells). Low expression levels were detected in the pleomorphic adenoma samples and adenoid cystic carcinomas without metastasis and recurrence, whereas higher expression levels were observed in the adenoid cystic carcinoma with metastasis and recurrence (P<0.05). This result indicates that NOTCH1 might play an important role in the metastasis of adenoid cystic carcinoma.

**Table 1:** The expression of NOTCH1 in normal salivary tissues, pleomorphic adenoma tissues and SACC samples

Samples	Cases	Negative	Positive	Strong Positive	P value
Normal Salivary	10	7	3	0	
Pleomorphic adenoma	22	6	15	1	0.07^a^
SACC	25	2	12	11	<0.01^b^
Without Metastasis	14	2	9	3	0.02^c^
With Metastasis	11	0	3	8	0.03^d^

a. When compared with normal salivary

b. When compared with normal salivary, P=0.0004; when compared with Pleomorphic adenoma, P=0.0052

c. When compared with normal salivary

d. When compared with SACC without metastasis

### NOTCH1 promotes cell proliferation *in vitro*

**Figure 2:**
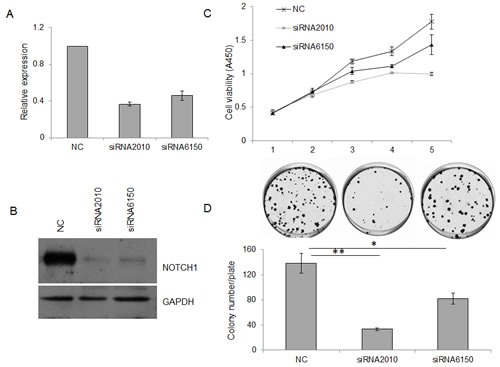
Knockdown of NOTCH1 inhibits the proliferation of SACC-83 cells A-B, 48 h after siRNA transfection, the expression of NOTCH1 in SACC-83 cells was measured by real-time PCR (A) and Western blot (B); C-D, After siRNA transfection, the proliferation of SACC-83 cells was detected by CCK-8 reagent (C, P<0.05 at day 3, and P<0.01 at days 4 and 5) and colony formation assay (D).

**Figure 3:**
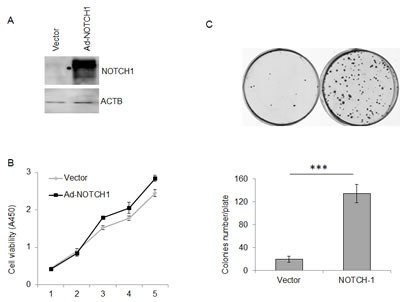
Overexpression of NOTCH1 promotes the proliferation of SACC-83 cells A, Western blot analysis of the overexpression of NOTCH1 in SACC-83 cells infected with adenoviral vector at an MOI of 5-10; B-C, After adenoviral infection, the proliferation of SACC-83 cells was detected by CCK-8 reagent (B, P<0.05 at days 3, 4 and 5) and colony formation assay (C, P<0.01, n=3).

To investigate the effect of NOTCH1 on the proliferation of cancer cells, siRNA-mediated knockdown of NOTCH-1 was employed in SACC-83 cells. As expected, both siRNAs targeting NOTCH1 (siRNA-2010 and siRNA-6150) efficiently reduced NOTCH1 expression in the SACC-83 cells compared with the negative control (NC), as shown by real-time RT-PCR (Fig. 2A) and Western blot (Fig. 2B). Additionally, these siRNAs significantly inhibited the growth of the SACC-83 cells, as measured using the CCK8 reagent (Fig. 2C, P<0.05 at day 3, and P<0.01 at days 4 and 5) and colony formation assays (Fig. 2D, P<0.05, n=3). To further verify the results of this loss-of-function study, SACC-83 cells were infected with a recombinant adenovirus (Ad-NOTCH1) carrying the correct coding sequence of the intracellular cytoplasmic domain of NOTCH1. Overexpression of NICD1 in SACC-83 cells (Fig. 3A) slightly increased cell proliferation over the short term as detected by the CCK8 assay (Fig. 3B, P<0.01 at days 3 and 5) but robustly increased cell proliferation over the long term as measured by colony formation assay (Fig. 3C, P<0.001). These collective data imply that overexpression of NOTCH1 promotes SACC proliferation *in vitro*, supporting the oncogenic roles of NOTCH1 in SACC.

### NOTCH1 increases cell migration and invasion *in vitro*

**Figure 4:**
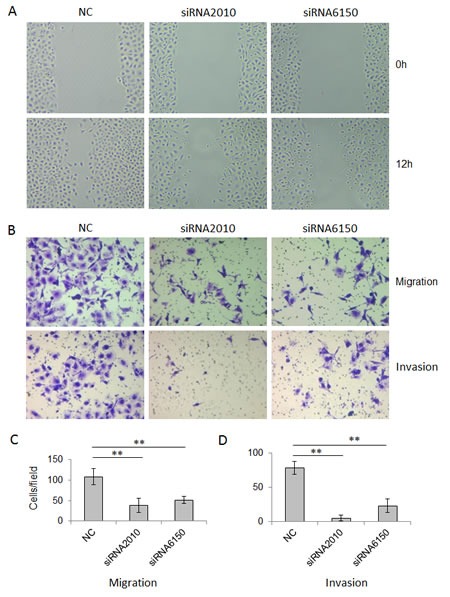
Knockdown of NOTCH1 inhibits the migration and invasion abilities of SACC-83 cells A, A photomicrograph of scratch wounds made in the siRNAs transfected SACC-83 cell layer shows inhibited cellular motility in the NOTCH1 silenced cells compared with the control. B, Representative images of the transwell assay without (upper panel) or with (lower panel) coated Matrigel after siRNA transfection. C, The number of cells that migrated through uncoated filters (i.e., no Matrigel), which represents the migratory ability of SACC-83 cells. D, The number of cells that were able to pass through filters precoated with Matrigel, which represents the invasive ability of SACC-83 cells. The counts of the cells are presented as the mean values per field from at least five randomly selected low-powered fields (x200) from three independent experiments (error bars, means ± SD). P<0.01 when compared with the control (NC).

**Figure 5:**
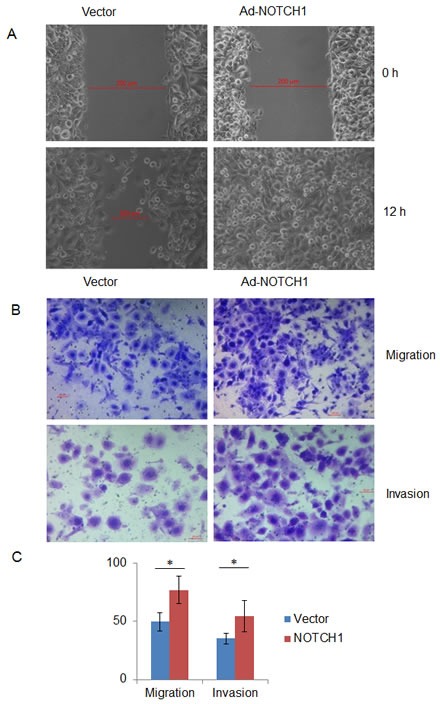
Overexpression of NOTCH1 increases the migration and invasion abilities of SACC-83 cells A, A photomicrograph of scratch wounds made in the infected SACC-83 cell layer shows enhanced cellular motility in the adenovirus Ad-NOTCH1-infected cells compared with the control adenovirus Ad-GFP infection; B, Representative images of the transwell assay without (upper panel) or with (lower panel) coated Matrigel after adenoviral infection; C, The number of cells that migrated or passed through the filters was counted. The counts of the cells are presented as the mean values per field from at least five randomly selected low-powered fields (x200) from three independent experiments (error bars, means ± SD). P<0.05 when compared with the control (vector).

Next, we asked whether NOTCH1 plays a role in the migration and invasion of SACC. As shown in Fig. 4, the knockdown of NOTCH1 in SACC-83 cells significantly inhibited cell migration (Fig. 4A, B and C, P<0.01, n=3) and invasion (Fig. 4B and C, P<0.01, n=3). In contrast, the overexpression of NICD1 in SACC-83 cells promoted cell motility, as indicated by the wound healing assay (Fig. 5A) and transwell assay (Fig. 5B and C, P<0.05, n=3), as well as cell invasiveness (Fig. 5B and C, P<0.05, n=3). These results confirm that NOTCH1 is an oncogene in SACC that may contribute to the migration and invasion of adenoid cystic carcinoma cells.

### Knockdown of NOTCH1 inhibits tumorigenicity *in vivo* and induces apoptosis

**Figure 6:**
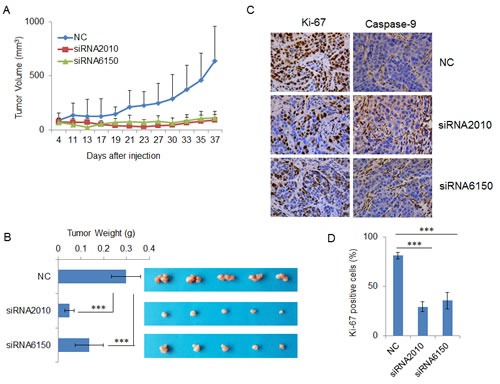
Knockdown of NOTCH1 inhibits tumorigenicity *in vivo* and induces apoptosis A-B, After transfection with siRNAs, SACC-83 cells were subcutaneously injected into the flanks of nude mice (n = 5), tumor sizes were measured using a digital caliper twice per week (A) and the tumors were excised, photographed and weighed (B); C, The expression of Ki-67 (left panel) and cleaved Caspase-9 (right panel) were detected in the xenograft tumors using immunohistochemistry (DAB, 400X).

To further address the oncogenic effect of NOTCH1 on tumorigenicity *in vivo*, endogenous NOTCH1 was silenced by transfecting NOTCH1-specific siRNAs into SACC-83 cells. The transfected cells were then inoculated subcutaneously into the flanks of athymic mice. Intriguingly, NOTCH1 knockdown inhibited tumor growth, as the tumor size under observation (Fig. 6A and B) and the wet weight (Fig. 6B) of xenograft tumors formed from cells transfected with both siRNA2010 and siRNA6150 were significantly decreased compared with that of the control. Next, we detected the expression of Ki67, and Caspase-9 in the xenograft tumors using immunohistochemistry. After the knock down of NOTCH1 in SACC-83 cells, the percentage of proliferating cells decreased and that of apoptotic cells increased (Fig. 6C and D), which indicated that downregulation of NOTCH1 in SACC cells can inhibit cell growth by inducing cellular apoptosis.

### NOTCH1 regulates cellular apoptosis via apoptosis-related gene expression

**Figure 7:**
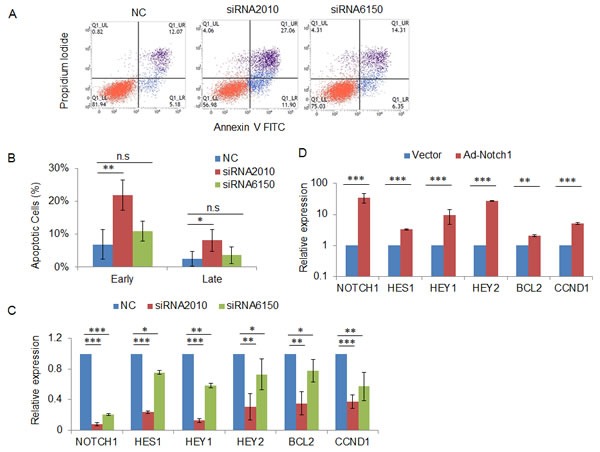
Knockdown of NOTCH1 induces cell apoptosis via regulation of the expression of apoptosis-related genes A, 48 h after transfection with the indicated siRNAs, SACC-83 cells were stained with Annexin V and propidium iodide, and the apoptotic cells were analyzed by flow cytometry; B, The percentage of early apoptosis cells (Annexin V-positive and PI-negative) and late apoptosis cells (Annexin V positive and PI positive). The data represent the results of three independent experiments. C-D, 48 h after transfection with the indicated siRNAs (C) or infection with NOTCH1 overexpression adenovirus (D), the expression of the NOTCH1 target genes HES1, HEY1, HEY2, BCL-2, and CCND1 was measured by real-time RT-PCR.

To validate our findings in xenograft tumors, we detected apoptotic cells using Annexin V and PI staining and flow cytometric analysis after transfection of NOTCH1 siRNAs in SACC-83 cells. The results showed that 48 h after transfection, the percentages of both early apoptosis cells (Annexin V-positive and PI-negative) and late apoptosis cells (Annexin V-positive and PI-positive) were higher in NOTCH1-silenced cells compared with those of the negative control cells (Fig. 7A and B). To further explore the underlying molecular mechanisms, we measured the expression of known NOTCH1 target genes using qRT-PCR. The results showed that knockdown of NOTCH1 in SACC-83 cells inhibited the expression of HES1, HEY1, HEY2, BCL2 and CCND1 (Fig. 7C), whereas overexpression of NOTCH1 increased the expression of these genes (Fig. 7D).

## DISCUSSION

Notch activity regulates tumor biology in a complex, context-dependent manner. Both the upregulation and downregulation of NOTCH1 have been observed in human cancers when compared with normal samples as shown by many study (for review see ref [2-3]) and the Oncomine database (Fig S1). Similar to its expression pattern, NOTCH1 has been shown to either promote or suppress tumor genesis, growth, and metastasis through its regulation of different target genes in a specific tissue environment and cancer microenvironment. Our data from KM Plotter also demonstrated that higher expression of NOTCH1 results in poor recurrence-free survival in breast cancer but better overall survival in lung cancer. Our results revealed that NOTCH1 was upregulated in SACC tissues when compared with normal tissues, and this upregulation was even higher in SACC tissues with metastasis and recurrence when compared with SACC tissues without metastasis (Fig. 1C and Table 3), indicating that NOTCH1 might play an oncogenic role in the tumorigenesis and metastasis of SACC.

The role of NOTCH1 in cellular proliferation and apoptosis has been deciphered in many cell types. In a limited number of tumor types, including human hepatocellular carcinoma and small cell lung cancer [22, 23], NOTCH1 plays an antiproliferative role. However, in most studies, NOTCH1 has demonstrated oncogenic roles. Li found that downregulation of NOTCH1 expression could suppress the proliferation and induce the apoptosis of U373MG and SHG44 glioblastoma cells [24]. It was also reported that aberrant NOTCH1 activation can induce BCL-2 overexpression and increase cell survival, and the noncanonical activation of NOTCH1 by membrane type matrix metalloproteinase sustains melanoma cell growth [25]. In the present study, we found that overexpression of NOTCH1 in SACC cell promotes cell growth and knockdown of NOTCH1 inhibits cell proliferation *in vitro* and tumorigenicity *in vivo* by inducing cell apoptosis.

Notch signaling is highly context- and cell type-dependent, although certain genes are consistently upregulated by activated Notch across many tissue types. In this study, we examined the well-known Notch target genes in SACC cells and found that HES1, HEY1, HEY2, BCL-2, and CCND1 were upregulated by overexpression of activated Notch and downregulated by silenced NOTCH1 (Fig. 7C and D), but there were no changes in the expression of ID4, HES5, PAX6, SOX9, MYC, and CCND3 (data not shown). Among these validated target genes, BCL-2 is a well-known anti-apoptotic gene [26], and CCND1 is a cell cycle-related gene [27], consistent with the increased apoptotic cells (Fig. 6C and Fig. 7A and B) and decreased Ki67-positive cells (Fig. 6C) following knockdown of NOTCH1. Additionally, HES1, HEY1, and HEY2 have been reported as proliferation- and apoptosis-related genes. Abdei Aziz [28] found that when NOTCH1 and its target genes Hes1 were downregulated, the HepG2 cells showed significant decrease in cell proliferation rate. Moriyama et al [29] provided evidence that Notch signaling, acting through HES1, plays an important role in the survival of immature melanoblasts by preventing cell apoptosis. Li [30] indicated that Notch1 mediates smooth muscle cells proliferation through HEY2.

Metastasis and invasion are two important factors that affect the prognosis and recurrence of SACC patients. Our results also showed that NOTCH1 can promote cell migration and invasion in SACC cells (Fig. 4 and 5), although the underlying molecular mechanism remains unclear. It was found that downregulation of NOTCH1 can decrease the migration and invasion of HCC cells by regulating E-cadherin and CD44v6 [31]. Luo found that expression levels of NICD and p21 were associated with tumor invasion in gastric cancer [32]. Further investigations are warranted to more precisely determine the molecular mechanism of NOTCH1 in the invasion and metastasis of SACC. The results of the present study suggest that NOTCH1 inhibitors may have potential therapeutic applications in treating SACC patients by inhibiting cell growth and metastasis.

## MATERIALS AND METHODS

### Cell culture and clinical samples

The SACC-83 cell line was obtained from the Peking University Health Science Center. The cells were maintained in RPMI-1640 (Gibco BRL, Grand Island, NY) with 10% fetal bovine serum (Gibco) and incubated in a humidified atmosphere of 95% air and 5% CO_2_ at 37°C. Experiments were performed using cells in the exponential phase of growth. Tissue samples were obtained from the First Affiliated Hospital of the Fujian Medical University and the Fujian Medical University Union Hospital. Ten normal salivary tissues, 22 pleomorphic adenoma tissues and 25 SACC samples (11 cases with metastasis and recurrence and 14 cases without metastasis and recurrence) were included. This study was approved by the Institutional Review Board of Fujian Medical University, and written informed consent was obtained from each participant.

### RNAi transfection

The negative control (NC) siRNA and two siRNAs against NOTCH1 were synthesized (GenePharma, Shanghai, China). The siRNA sequences are listed in Table 2. Cells were transfected with siRNAs using Lipofectamine RNAiMAX (Invitrogen, USA) according to the manufacturer's instructions.

**Table 2:** The siRNA sequences

Name	Sequence
siRNA-NOTCH1-6150	5′-GGCUAACAAAGAU AUGCATT-3′
	5′-GCAUAUCUUUGUU AGCCCTT-3′
siRNA-NOTCH1-2010	5′-CAGGGAGCAUGUGU AACAUTT-3′
	5′-AUGUUACACAUGCU CCCUGTT-3′
NC	5′-UUCUCCGAACGUGU CACGUTT-3′
	5′-ACGUGACACGUUCG GAGAATT-3′

### Construction of the NOTCH1 adenoviral vector and infection

The DNA fragment of NICD1 was amplified from the NICD1 plasmid (a kind gift from Dr. Glenn Doughty, Harvard Medical School) [14] by PCR with high-fidelity Platinum Taq DNA Polymerase (Invitrogen). The paired primers were 5′-AAGGAAAAAAGCGG CCGCAGATGCGG CGGCAGCATGGC-3′ and 5′- CCGCTCGAGCTTGAAGGCCTCCGGAAT -3′. The fragment was then inserted into the Not I and Xho I cloning sites of the pShuttle-IRES-hrGFP-1 vector (Stratagene, USA). After verification by sequencing, BJ5183 cells (Stratagene, USA) were transformed with the linearized shuttle vector containing the gene of interest. A recombination event in the bacterial cells resulted in the production of recombinant adenovirus carrying the NICD1 fragment. The adenovirus was packaged and amplified in AD-293 cells (Stratagene, USA). After production, the adenovirus was amplified for three rounds. Adenovirus-GFP (Vector) was constructed and used as the control. SACC-83 cells were infected with the adenoviral vectors at the same multiplicity of infection (MOI) of 10.

### Immunohistochemistry

For the immunohistochemical assays, 5-μm-thick tissue sections were mounted on slides coated with poly-L-lysine. After deparaffinization in xylene, the sections were rehydrated in a decreasing gradient of ethanol and washed for 10 min in phosphate-buffered saline (PBS) (pH 7.2). Endogenous peroxidase activity was inhibited by incubation in methanol containing 3% H_2_O_2_ for 10 min. After several washes in PBS, the sections were blocked with a universal blocking reagent (Maxin, USA) for 10 min at room temperature and then incubated with the primary antibody against NOTCH1 (1:200, R&D, USA), Ki67 (1:500, ABCAM, UK), or Caspase-9 (1:200, ABCAM, UK) for 1 h at room temperature. After several washes in PBS, the sections were incubated with a biotin-conjugated secondary antibody (Maxin) for 10 min at room temperature. After several washes in PBS, the sections were incubated with streptavidin-peroxidase (Maxin) for 10 min at room temperature. The sections were rinsed with PBS, and the antibody complexes were visualized by incubation with diaminobenzidine tetrahydrochloride (DAB) chromogen (Maxin). The sections were then counterstained with hematoxylin (Dako, Denmark), dehydrated, and examined by light microscopy. All slides were reviewed independently by two pathologists who were blinded to each other's readings. The staining results were assessed on a three-tier scale: negative indicated no staining, 1+ indicated weak staining and 2+ indicated strong staining. Immunohistochemical results were graded with 3 different scores (negative, positive and strong positive) as follows: negative indicated no staining or 1+ staining in ≤30% of cells, positive indicated 1+ staining in >30% of cells or 2+ staining in <50% of cells and strong positive indicated 2+ staining in >50% of cells.

### Quantitative real-time PCR analysis

Total RNA was extracted from cells with Trizol reagent (Invitrogen, USA) and reverse-transcribed into cDNA with the PrimeScript RT reagent kit (TaKaRa, Japan). The cDNA was used as the template to detect the expression of the genes of interest by qRT-PCR with SYBR Premix Ex Taq^TM^ (TaKaRa, Japan). The primers used in this study are listed in Table 3. Data were analyzed according to the 2^−Δ ΔCt^ method [33].

**Table 3:** Real-time PCR primers used in this study

Gene	Accession no.	Forward	Reverse
ACTB	NM001101.3	GACAGGATGCAGAAGGAGATCA	TTTTAGGATGGCAAGGGACTTC
NOTCH1	NM017617.3	GGAAGTTGAACGAGCATAGTCC	GCATGATGCCTACATTTCAAGA
Hey1	NM012258.3	CGAGGTGGAGAAGGAGAGTG	CTGGGTACCAGCCTTCTCAG
Hes1	NM005524.3	AGGCGGACATTCTGGAAATG	CGGTACTTCCCCAGCACACTT
Hey2	NM012259.2	GAACAATTACTCGGGGCAAA	TCAAAAGCAGTTGGCACAAG
CCND1	NM053056.2	CCCCGCACGATTTCATTGAACA	CATGGAGGGCGGATTGGAAATG
BCL2	NM000633.2	CATGTGTGTGGAGAGCGTCAAC	GGAGAAATCAAACAGAGGCCGC
GAPDH	NM002046.5	TGCACCACCAACTGCTTAGC	AGCTCAGGGATGACCTTGCC

### Western blot assay

Total protein was separated by 8% SDS-PAGE and transferred onto PVDF membranes (Amersham, USA). Subsequently, the membranes were immunoblotted with primary antibodies against NOTCH1 (1:1000 dilution, Cell Signaling, USA) or GAPDH (Abmart, USA) in 5% bovine serum albumin overnight, washed three times with tris-buffered saline with 0.1% Tween20, and incubated with secondary antibody (1:2000 dilution, Abcam, USA). The immunoreactive protein bands were visualized using CDP STAR reagent (Roche, IN, USA), and signals were scanned with a densitometer for semi-quantification of the signal intensity.

### Cell viability assay

Cell proliferation was measured by counting viable cells with a Cell Counting Kit-8 (Dojindo, Kumamoto, Japan). Cells were first transfected with siRNA or infected with adenovirus for 24 h, then plated into a 96-well plate. At the same time each day for 5 consecutive days, the original culture medium was removed, and 10 μl cck8 and 90 μl fresh 1640 were added into each well. The cells were incubated at 37°C for 1 h. The absorbance of each well was measured with a microplate reader (Pharmacia Biotech, USA) at 450 nm.

### Colony formation assay

Twenty-four hours after siRNA transfection or adenovirus infection, the cells were plated into 6-cm plates (200 cells per plate) and cultured for 2 weeks (siRNA interference) or 10 days (adenovirus infection). Colonies were fixed with cold methanol for 10 min and stained with 1% crystal violet for 30 min.

### *In vitro* cell invasion assay

Cell invasion was determined using 24-well Matrigel-coated transwell chambers (8-μm pore size, BD Science, USA). Twenty-four hours after siRNA transfection or adenovirus infection, cells were serum starved for 24 h and then collected in 1640 containing 1% FBS. Cells were plated in the upper chamber at a density of 1.0×10^5^, and 800 μl of 1640 containing 10% FBS was added to the lower chamber. After incubation at 37°C for 48 h, the Matrigel and cells in the upper chamber were removed using a cotton swab and stained with 1% crystal violet for 10 min. Cells were counted and photographed by microscopy of at least five random fields (×200).

### *In vitro* cell migration assay

Cell migration assays were performed using 24-well transwell chambers (8- μm pore size, BD Science, USA). The procedure used for this assay was similar to that of the cell invasion assay, except that the transwell was not coated with Matrigel.

### Cell apoptosis assay

Cellular apoptosis was analyzed using the FITC Annexin V Apoptosis Detection Kit (BD Pharmingen^TM^, USA). At 48-h post-transfection, the cells were collected and washed in PBS, then stained with Annexin V and propidium iodide for 15 min. The percentage of apoptotic cells was quantified using a BD FACS Verse Flow cytometer.

### Xenograft cancer model

The experimental animal protocols were approved by the Animal Care and Use Committee of Fujian Medical University. Female BALB/c nude mice 6~8 week of age were purchased from the Center for Animal Experiments of Fujian Medical University. Prior to injection, 15 nude mice were assigned at random to three groups with five mice per group. Cells (2×10^6^) were suspended in 0.2 ml serum-free 1640 and injected into the right axillary fossa of each mouse. Tumor size was measured weekly and calculated using the formula V=width^2^×length/2. At the end of experiment, the tumors were harvested, washed once in PBS, and weighed.

### Statistical analysis

The statistical analysis of NOTCH-1 immunoreactivity was performed using the rank-sum test. The statistical analyses of PCR results and the *in vitro* cell migration/invasion assays were determined by Student's t-test. p<0.05 was considered statistically significant, and it was indicated in the figures as n.s when P>0.05, * when P<0.05, ** when P<0.01 and *** when P<0.001.

## SUPPLEMENTAL MATERIAL AND FIGURE


