# Supplementary material for: NOTCH1 signaling contributes to cell growth, anti-apoptosis and metastasis in salivary adenoid cystic carcinoma
Source: Oncotarget. 2014 Aug 6;5(16):6885–95. doi: 10.18632/oncotarget.2321 (PMC4196170; doi:10.18632/oncotarget.2321)
Supplement: Supplementary file 1 [file oncotarget-05-6885-s001.pdf]

NOTCH1 signaling contributes to cell growth, anti-apoptosis and metastasis in salivary adenoid cystic carcinoma

Supplementary Material

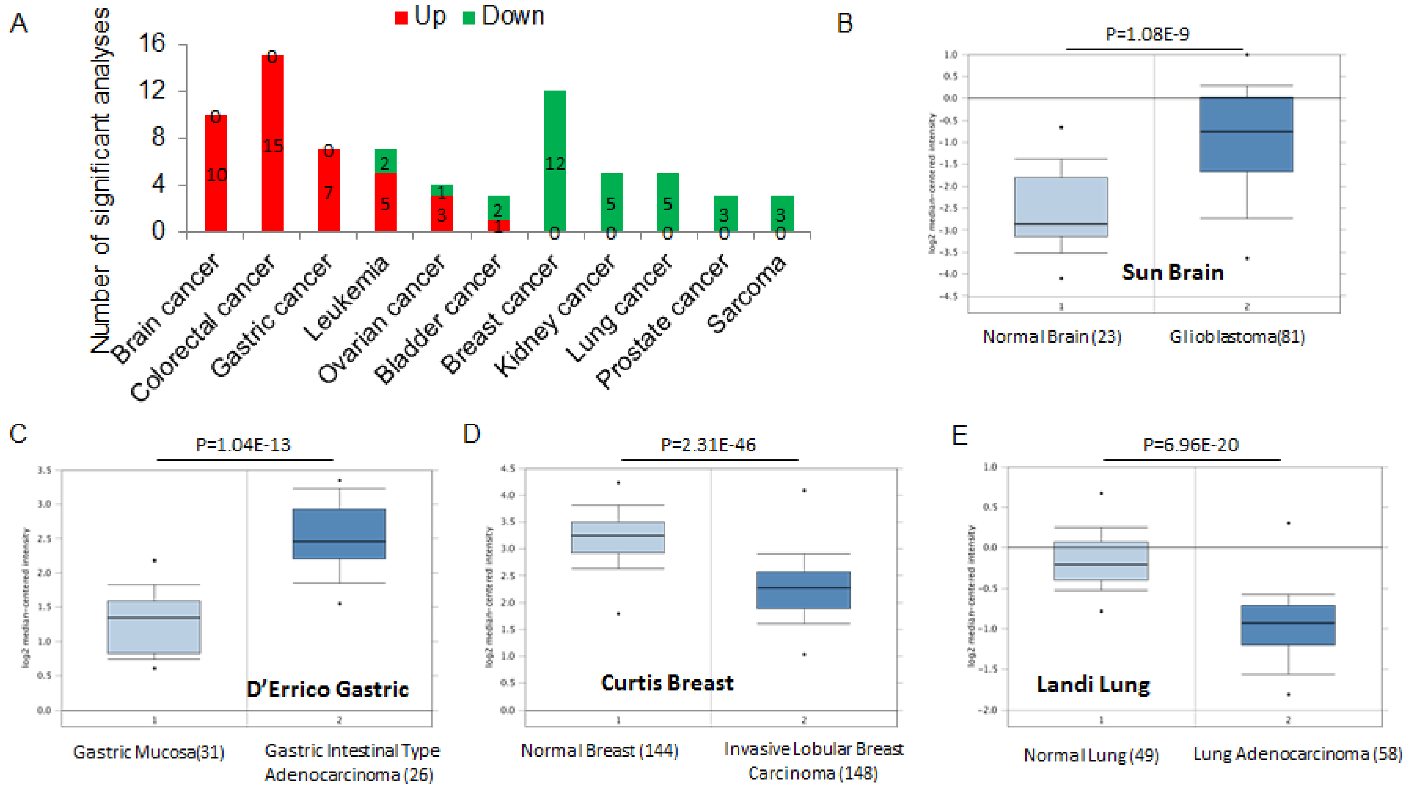

Fig S1: Deregulation of NOTCH1 in different types of cancer from Oncomine database. A, The number of analyses in different types of cancer in which NOTCH1 was upregulated (red) or downregulated (green). The threshold of P value was set as 0.01 and fold change was 1.5. B-E, Representative images of upregulation of NOTCH1 in the specified dataset of brain cancer (B), gastric caner (C) or downregulation in the dataset of breast cancer (D) and lung cancer (E).
